# Supplementary figures and images for: Removal of extracellular human amyloid beta aggregates by extracellular proteases in C. elegans
Source: eLife. 2023 Sep 20;12:e83465. doi: 10.7554/eLife.83465 (PMC10541181; doi:10.7554/eLife.83465)

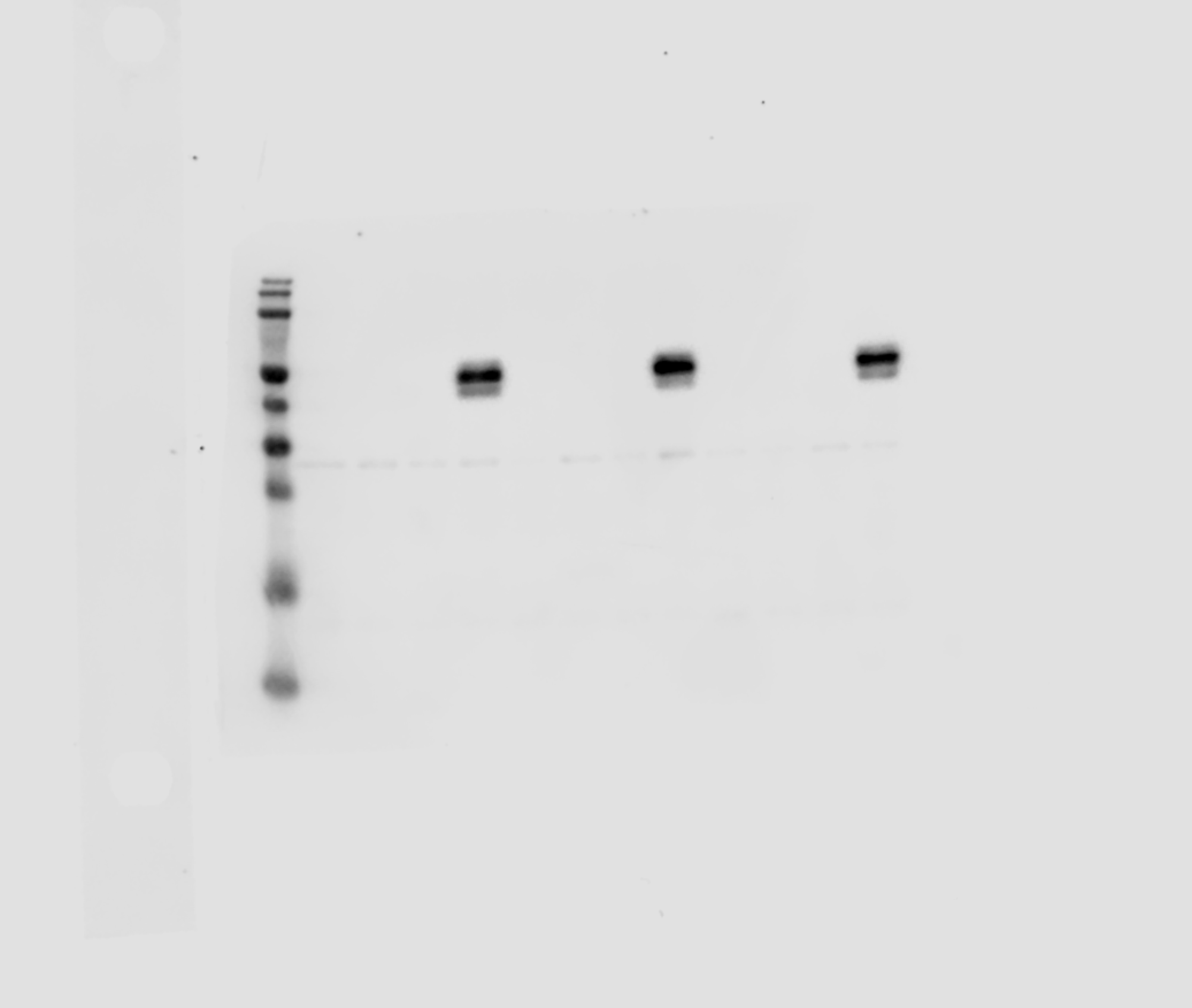

Supplement: Figure 1—figure supplement 1—source data 1. — Raw and labeled western blots for Figure 1—figure supplement 1. [file elife-83465-fig1-figsupp1-data1.zip › Abeta 6E10.tif]

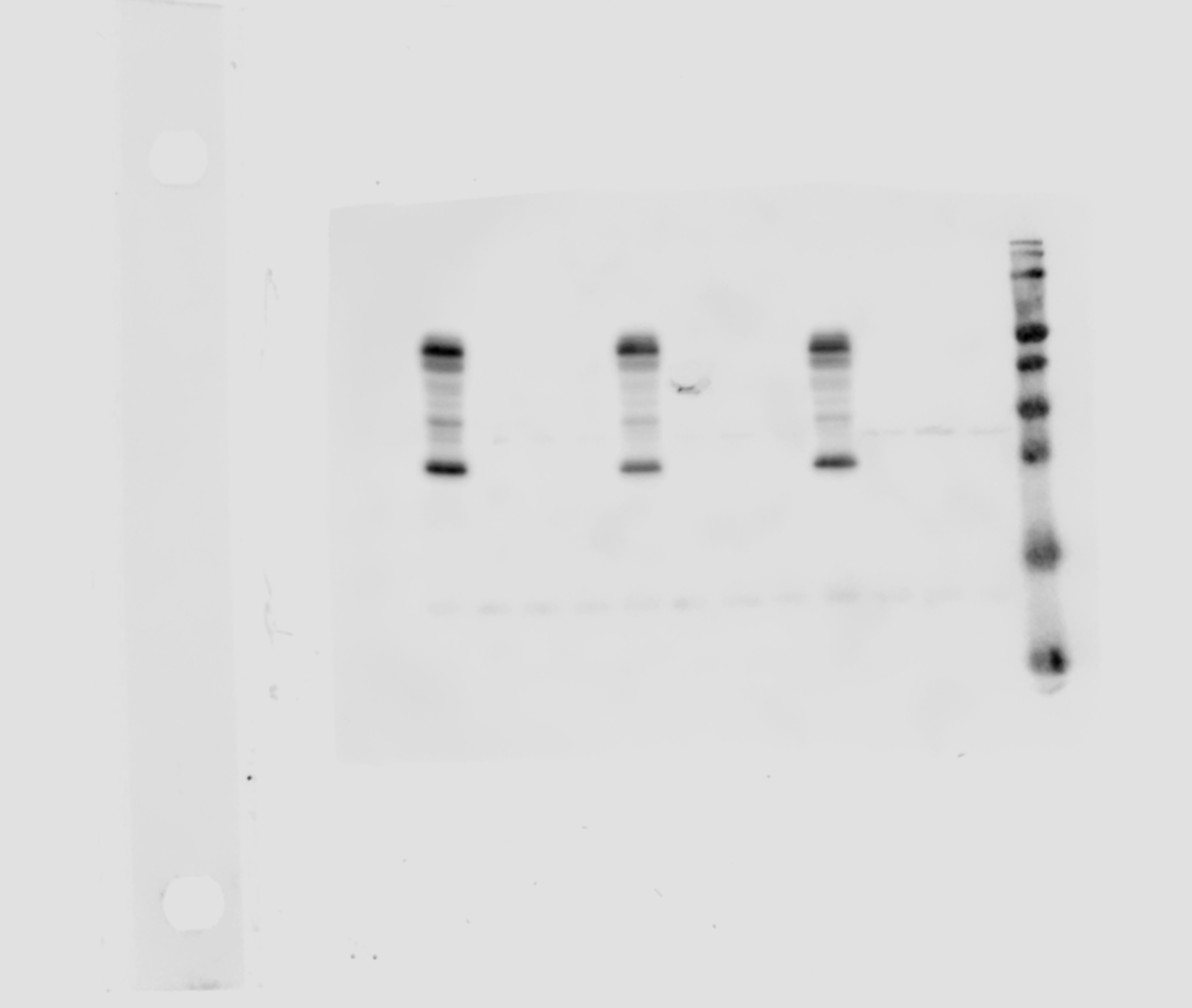

Supplement: Figure 1—figure supplement 1—source data 1. — Raw and labeled western blots for Figure 1—figure supplement 1. [file elife-83465-fig1-figsupp1-data1.zip › GFP.tif]

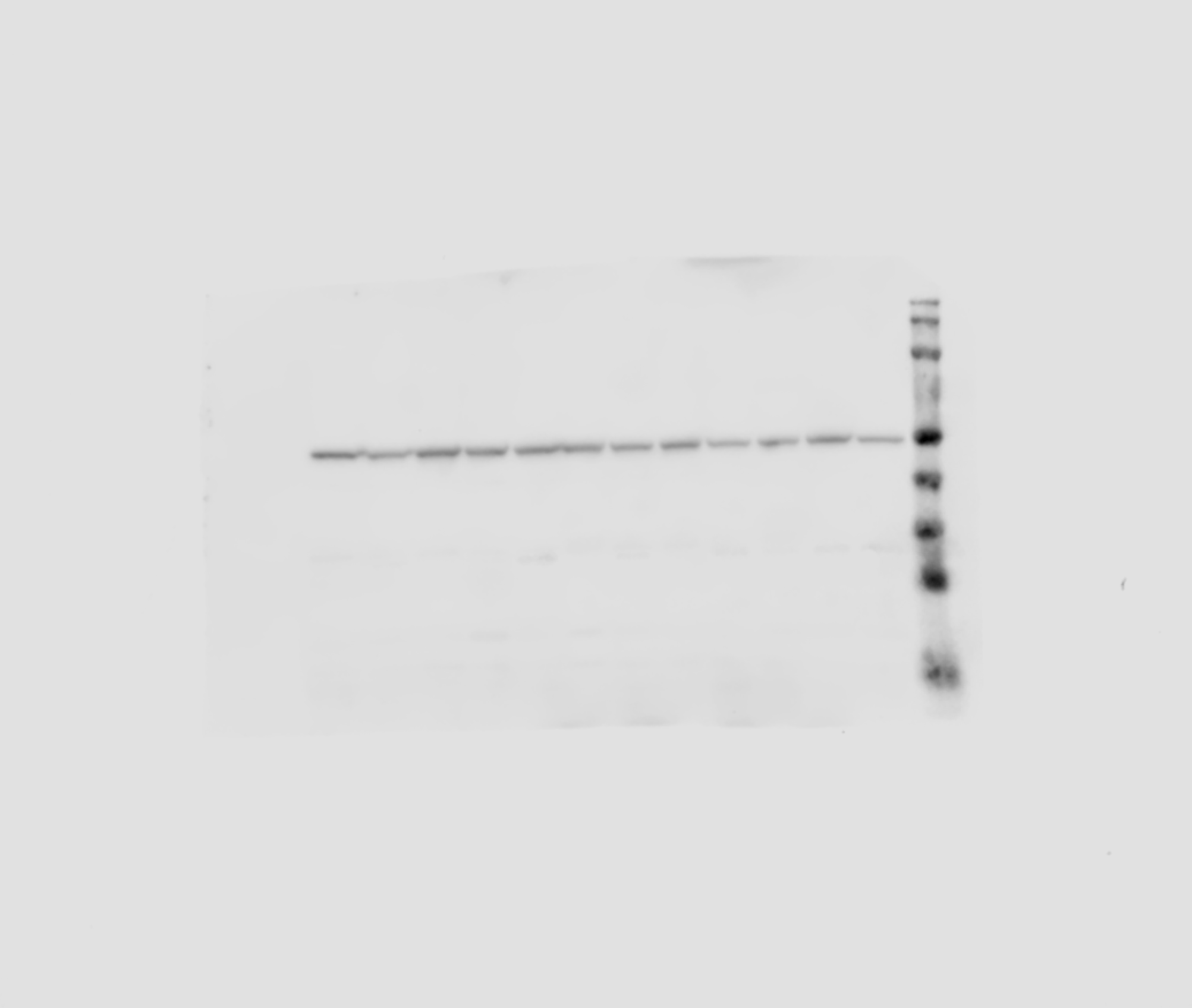

Supplement: Figure 1—figure supplement 1—source data 1. — Raw and labeled western blots for Figure 1—figure supplement 1. [file elife-83465-fig1-figsupp1-data1.zip › tubulin.tif]
